# Proteomic Analysis of *Listeria monocytogenes* FBUNT During Biofilm Formation at 10°C in Response to Lactocin AL705

**DOI:** 10.3389/fmicb.2021.604126

**Published:** 2021-01-29

**Authors:** Constanza Melian, Patricia Castellano, Franco Segli, Lucía M. Mendoza, Graciela Margarita Vignolo

**Affiliations:** Centro de Referencia para Lactobacilos, Consejo Nacional de Investigaciones Científicas y Técnicas (CERELA-CONICET), San Miguel de Tucumán, Argentina

**Keywords:** *Listeria monocytogenes*, biofilm control, cold temperature, proteins expression, lactocin AL705

## Abstract

*Listeria monocytogenes* is one of the major food-related pathogens and is able to survive and multiply under different stress conditions. Its persistence in industrial premises and foods is partially due to its ability to form biofilm. Thus, as a natural strategy to overcome *L. monocytogenes* biofilm formation, the treatment with lactocin AL705 using a sublethal dose (20AU/ml) was explored. The effect of the presence of the bacteriocin on the biofilm formation at 10°C of *L. monocytogenes* FBUNT was evaluated for its proteome and compared to the proteomes of planktonic and sessile cells grown at 10°C in the absence of lactocin. Compared to planktonic cells, adaptation of sessile cells during cold stress involved protein abundance shifts associated with ribosomes function and biogenesis, cell membrane functionality, carbohydrate and amino acid metabolism, and transport. When sessile cells were treated with lactocin AL705, proteins’ up-regulation were mostly related to carbohydrate metabolism and nutrient transport in an attempt to compensate for impaired energy generation caused by bacteriocin interacting with the cytoplasmic membrane. Notably, transport systems such as β-glucosidase IIABC (lmo0027), cellobiose (lmo2763), and trehalose (lmo1255) specific PTS proteins were highly overexpressed. In addition, mannose (lmo0098), a specific PTS protein indicating the adaptive response of sessile cells to the bacteriocin, was downregulated as this PTS system acts as a class IIa bacteriocin receptor. A sublethal dose of lactocin AL705 was able to reduce the biofilm formation in *L. monocytogenes* FBUNT and this bacteriocin induced adaptation mechanisms in treated sessile cells. These results constitute valuable data related to specific proteins targeting the control of *L. monocytogenes* biofilm upon bacteriocin treatment.

## Introduction

*Listeria monocytogenes* is the causative agent of the severe human and animal disease listeriosis, which has a high mortality rate (Listeriosis, 2017; Foodnet Centers for Disease Control and Prevention, 2018) and whose features include gastroenteritis in healthy individuals that can develop into a severe invasive illness in the elderly, pregnant women, infants, and immunocompromised (Ferreira et al., 2014). This foodborne pathogen is implicated in sporadic cases, outbreaks, and food recalls worldwide (Desai et al., 2019). The ability to grow under a wide range of environmental conditions, such as extreme pH values, low temperatures, and high salt concentrations, makes *L. monocytogenes* difficult to eradicate from food industrial facilities, persisting on equipment, utensils, floors, and drains, ultimately reaching food products by cross-contamination. Awareness about the presence of this bacterium at a retail level is relevant because it is the last step before food reaches the consumer (Gómez et al., 2015; Camargo et al., 2017; Jordan and McAuliffe, 2018). Ready-to-eat (RTE) foods are common vehicles involved in listeriosis outbreaks, this stimulating many countries to create specific legislation aimed at controlling *L. monocytogenes* in RTE foods after the 1990s (Camargo et al., 2017). The persistence of this pathogen in food environments is partly due to its ability to form biofilms; bacterial adhesion to industrial surfaces is of great concern as a chronic source of contamination (Bridier et al., 2015; Coughlan et al., 2016). There is abundant evidence indicating that pathogens capable of forming biofilm are self-protected from common cleaning procedures, allowing them to remain in the environment post-sanitation and increasing their resistance to antimicrobials/sanitizers (Bae et al., 2012; Rodríguez-López et al., 2018).

Strategies to overcome *L. monocytogenes*’ persistence through the inhibition of biofilm formation or removal of mature biofilms are therefore necessary. Innovative and eco-friendly approaches involving microorganisms and their metabolites, plant-derived compounds, *in situ* produced inhibitors, and bioregulation have recently emerged as biofilm control alternatives (Coughlan et al., 2016; Gray et al., 2018; Rodríguez-López et al., 2018). Moreover, transcriptomic and proteomic approaches were applied to identify genetic determinants and protein expression associated with *L. monocytogenes*’ ability to form biofilm, both as saprophyte in foods and in industrial premises (Barbosa et al., 2013; Tang et al., 2015; Lanciotti et al., 2019; Santos et al., 2019) and as an intracellular pathogen (Freitag et al., 2009). *L. monocytogenes*’ growth at low temperatures constitutes a high food safety challenge; cold stress adaptation mechanisms are therefore an essential skill enabling it to survive and disseminate in refrigerated products (Tasara and Stephan, 2006; Chan et al., 2007; Cacace et al., 2010; Piercey et al., 2016; Santos et al., 2019). In addition to a decreased metabolic capacity, to overcome the hurdle imposed by cold stress, *L. monocytogenes* have to increase the expression of genes involved in cell membrane function, nucleic acids, nutrients transport, cold shock proteins production, and macromolecular assemblies, such as ribosomes (NicAogáin and O’Byrne, 2016).

A wide range of lactic acid bacteria (LAB) produce bacteriocins, which are essentially active against the foodborne pathogen *L. monocytogenes* through ribosomally-synthesized peptides that kill target cells generally by increasing the permeability of the cytoplasmic membrane (Castellano et al., 2017). They can be used directly as a semi-purified compound or indirectly *via* the bacteriocin-producing organism (Castellano and Vignolo, 2006). Accordingly, the potential of bacteriocinogenic LAB and their bacteriocins as biosanitizers to prevent the attachment of *L. monocytogenes* to food facilities’ surfaces has been evaluated (Desai et al., 2012; Zhao et al., 2013; Pérez Ibarreche et al., 2016; Melian et al., 2019). In particular, *L. monocytogenes* biofilm treated with enterocin AS-48 (Caballero Gómez et al., 2013), nisin (García-Almendárez et al., 2008), and different *Lactobacillus* extracts (Winkelströter et al., 2015; Camargo et al., 2016) were evaluated. In a recent study, antilisterial lactocin AL705 was able to control *L. monocytogenes* FBUNT biofilm formation by a disruption of quorum sensing through a signal molecule inactivation; however, a lack of interference with autoinducer-2 (AI-2) signaling molecule was observed, suggesting that other molecule(s) different from AI-2 involved in biofilm formation were are the target of the bacteriocin (Melian et al., 2019). On this basis, the aim of this study was to analyze *L. monocytogenes* FBUNT proteome changes during biofilm formation at 10°C, exposed or not to lactocin AL705, by using a comparative proteomic approach.

## Materials and Methods

### Strains and Culture Conditions

*Listeria (L.) monocytogenes* FBUNT (Facultad de Bioquímica, Química y Farmacia, UNT, Argentina) was routinely cultivated in Tryptic Soy Both supplemented with 0.5% (w/v) of yeast extract (TSB-YE) for 16h at 30°C. *Lactobacillus* (*Lb*.) *curvatus* CRL1579, a derivative from the parental strain (*Lb. curvatus* CRL705 producer of two component bacteriocins lactocin 705 and anti-listerial bacteriocin lactocin AL705) able to produce only the bacteriocin lactocin AL705 (Castellano and Vignolo, 2006) was used in this study. The strain CRL1579 was grown in MRS broth at 30°C. Culture stocks were kept at −80°C in liquid media containing 15% (v/v) of glycerol. Unless otherwise stated, all the used media were supplied by Britania (Buenos Aires, Argentina).

### Bacteriocin Purification and Minimum Inhibitory Concentration

One liter from an overnight culture of *Lb. curvatus* CRL1579 in MRS broth was centrifuged (11,000*g*, 20min at 4°C) (Avanti J-25I centrifuge, Beckman, United States) to remove bacterial cells. The bacteriocin was precipitated from the supernatant with 60% saturated ammonium sulphate (Cicarelli-Reagents S.A); the purification was performed following the protocol described by Melian et al. (2019). Antimicrobial activity of the semi-purified bacteriocin solution was determined by a well diffusion assay according to Castellano et al. (2004). To determine the MIC of lactocin AL705 against *L. monocytogenes* FBUNT, Mueller-Hinton broth micro-dilution method following the Clinical and Laboratory Standards Institute (CLSI) 2017 guidelines assay was used. A 0.5 McFarland-standardized *L. monocytogenes* inoculum was used to prepare a total volume of 200μl in each well of the microplate. After incubation for 20h at 30°C, optical density at 600nm (OD_600_) readings were carried out using a VersaMax microplate reader (Molecular Devices, Sunnyvale, CA, United States). The MIC was defined as the lowest concentration of lactocin AL705 that produced at least 95% growth reduction. The growth inhibition percentages at different bacteriocin concentrations for *L. monocytogenes* FBUNT were calculated as % growth inhibition = [1-(Ac/A0)] × 100, where Ac represents the absorbance of the well with a bacteriocin concentration c, and A0 is the absorbance of the control well (without bacteriocin). In addition, antilisteria activity was determined by the critical dilution assay according to Castellano et al. (2004), as arbitrary units per milliliter (AU/ml), defined as the reciprocal of the highest dilution that presented an inhibition zone.

### Sessile and Planktonic Cells Collection

Biofilm development by *L. monocytogenes* FBUNT was evaluated as reported by Pérez Ibarreche et al. (2016) with minor modifications. In brief, 2ml of TSB-YE medium was added to each well of 24-wells polystyrene microplates. Overnight culture of *L. monocytogenes* FBUNT (1% v/v) was inoculated on the microplates in the presence or absence of bacteriocin. A sub-inhibitory concentration (20AU/ml) of lactocin AL705 was used according to Melian et al. (2019). Microplates were statically incubated over 6days at 10°C (temperature used in meat processing). Then, wells were washed with phosphate-buffered saline (PBS) to remove non-adherent cells and the attached (biofilm) cells were resuspended in 100μl of PBS, centrifuged, and the obtained pellet was stored at −80°C until use. In addition, tubes with 3ml of sterile TSB-YE medium were inoculated with an overnight culture of *L. monocytogenes* FBUNT (1%) to obtain planktonic cells after 6days at 10°C. The tubes were incubated under the same conditions as sessile cells, centrifuged, and the pellet kept at −80°C. Three independent cultures (biological replicates) were undertaken for both planktonic and sessile conditions.

### Quantitative Proteomic

#### Protein Extraction

Total protein extraction was carried out for planktonic, biofilm, and biofilm AL705-treated cells from three independent cultures. Cells were suspended in a lysis buffer (Tris-HCl 20mM, pH 7.6, NaCl 10mM, SDS 0.5mM, PMSF 1mM) and broken using a BeadBeater (MiniBeadBeater-16, BioSpec, Bartlesville, OK) with 7cycles of 1min, allowing to stand for 1min on ice between each cycle. Unbroken cells and cell debris were removed by centrifugation (10,000*g*, 30min at 4°C). Protein concentration of cell free supernatants was determined by Bradford assay. Extracts were stored at −80°C for further use.

#### Protein Analysis

Equal amounts of total proteins (100μg) of each sample were used for electrophoretic run. Gels of Tris-tricine (15% as separator gel and 5% as concentrator gel) were used for SDS-PAGE. A voltage of 55mA was applied to the gel using a Mini-PROTEAN Tetra Cell Vertical Electrophoresis unit (Bio-Rad) until migration of 1cm. Gels were stained with Coomassie Safe (Bio-Rad) according to the instructions of the manufacturer and distained with distilled water. Bands were cut and stored until further protein digestion. Three technical replicates for each biological sample were performed. Protein digestion and Mass Spectrometry (MS) analyzes were performed at the Proteomics Core Facility of CEQUIBIEM (University of Buenos Aires-CONICET). Proteins were digested with trypsin (Promega V5111) and peptides were purified and desalted with ZipTip C18 columns (Millipore). Digests were analyzed by nanoLC-MS/MS in a Thermo Scientific QExactive Mass Spectrometer coupled to a nanoHPLC EASY-nLC 1000 (Thermo Scientific). For LC-MS/MS analyzes, approximately 1μg of peptides was loaded onto the column and eluted for 120min using a reverse phase column (C18, 2μm, 100A, 50μm × 150mm) Easy-Spray Column PepMap RSLC (P/N ES801) suitable for separating protein complexes with a high-resolution degree. The flow rate used for the nano column was 300nl/min and solvent range was from 7% solvent B (5min) to 35% solvent A (120min). Solvent A was 0.1% formic acid in water whereas solvent B was 0.1% formic acid in acetonitrile and the injection volume was 2μl. The MS equipment has a high collision dissociation cell (HCD) for fragmentation and an Orbitrap analyzer (Thermo Scientific, Q-Exactive) according to the protocol described by La Greca et al. (2018).

#### Analysis of MS Data

Data were processed using Proteome Discoverer and Perseus software. A protein database of *Listeria monocytogenes* EGD-e (ATCC BAA-679) was used for peptide identification. Proteome Discoverer calculated an area for each protein in each condition using the area under the curve of the 3 most intense peptides for a protein. Areas were calculated for each of the three biological triplicates and later normalized. The data obtained for each protein area were processed with the Perseus program (Max Planck Institute of Biochemistry, 1.5.5.3 version) that allows for a deeper statistical analyzes. Different scatter plots were done according to the compared samples. For each couple of samples, we plotted Log *p*-value (-Log Student *T*-test *p*-value A_B) on the y-axis vs. Student *T*-test Difference A-B in x-axis. Proteins that appear in the volcano plot with a fold change greater than two (less than −1 or greater than 1 on the x-axis of the graph) and a *p*-value below 0.05 (above 1.3 on the y-axis of the graph) were considered as differentially expressed (La Greca et al., 2018). In addition, proteins that were exclusively detected in one growth condition, meaning their comparison between samples was not possible, were designated by the used program as NAN (Non Assigned Number); in this study they correspond to those exclusively expressed in lactocin AL705-treated biofilm condition.

#### Bioinformatics Analysis

The Venn diagram was performed using the jvenn online tool (Bardou et al., 2014). The functional classification of the identified proteins was performed using the databases Universal Protein Resource (UniProt) and the eggnog online framework to identify the clusters of orthologous groups (COGs) (Huerta-Cepas et al., 2016). Protein-protein interactions were investigated *via* the STRING database using the accession numbers of all differentially expressed proteins (Szklarczyk et al., 2015).

#### Data Availability

The mass spectrometry proteomics data have been deposited to the ProteomeXchange Consortium *via* the PRIDE partner repository with the dataset identifier PXD022168.

## Results

### Quantitative Analysis of Proteins During *L. monocytogenes* Biofilm Formation

**Figure 1:**
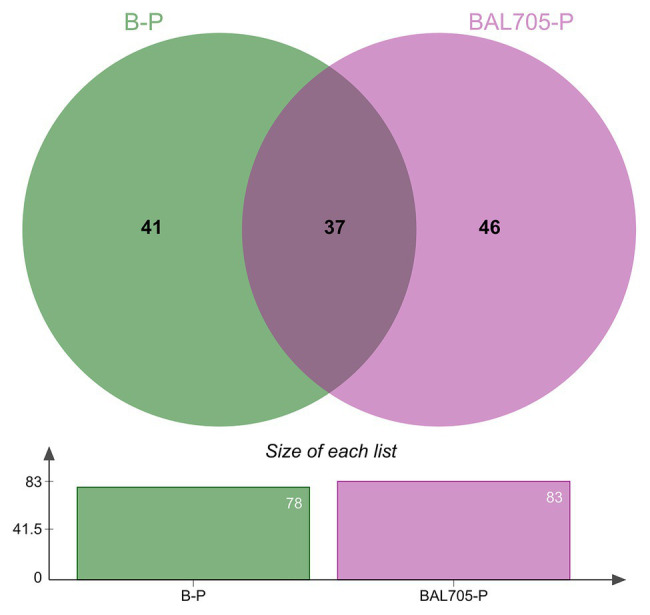
Venn diagram of the distribution of unique and shared differentially expressed proteins identified in biofilms formed by *L. monocytogenes* in the absence (B-P) and presence of lactocin AL705 (BAL705-P) at 10°C in comparison to planktonic cells grown at 10°C without lactocin.

The analysis of *L. monocytogenes* FBUNT proteome was performed in order to elucidate the regulation of proteins during biofilm formation at 10°C as well as the effect of lactocin AL705. In accordance with a previous study, the calculated MIC of lactocin AL705 against *L. monocytogenes* FBUNT was 40AU/ml (7.4μg/ml), whereas the subinhibitory concentration of 20AU/ml caused the highest inhibition of biofilm (75%) but had no effect on the growth of the planktonic population (Melian et al., 2019). The *L. monocytogenes* FBUNT proteome was made up of 1,011 proteins representing 35.4% of the strain reference (EGD-e) proteome. The proteins that showed >2-fold change (*p* ≤ 0.05) were considered as significantly differentially expressed. As is shown in the Venn diagram (Figure 1), a total of 124 proteins modified their abundance in lactocin-treated and untreated biofilm cells regarding the planktonic state. Of these, 87 proteins specifically changed their expression during biofilm formation in the presence or absence of bacteriocin, while 37 were shared between both conditions. The number of proteins detected uniquely in the comparison between the lactocin-treated sessile cells and the untreated planktonic cells was slightly higher.

Proteins were classified according to their function in the following 19 functional categories: amino acid, carbohydrate, coenzyme, lipid, nucleotide, and vitamin metabolism, cell cycle control, cell wall biogenesis, defense mechanisms, energy production, intracellular trafficking, motility, oxidoreductase activity, post-translation modification, replication, signal transduction mechanisms, transcription, translation, transport, and uncharacterized.

### Up-Regulated Proteins by *L. monocytogenes* FBUNT Sessile Cells

**Figure 2:**
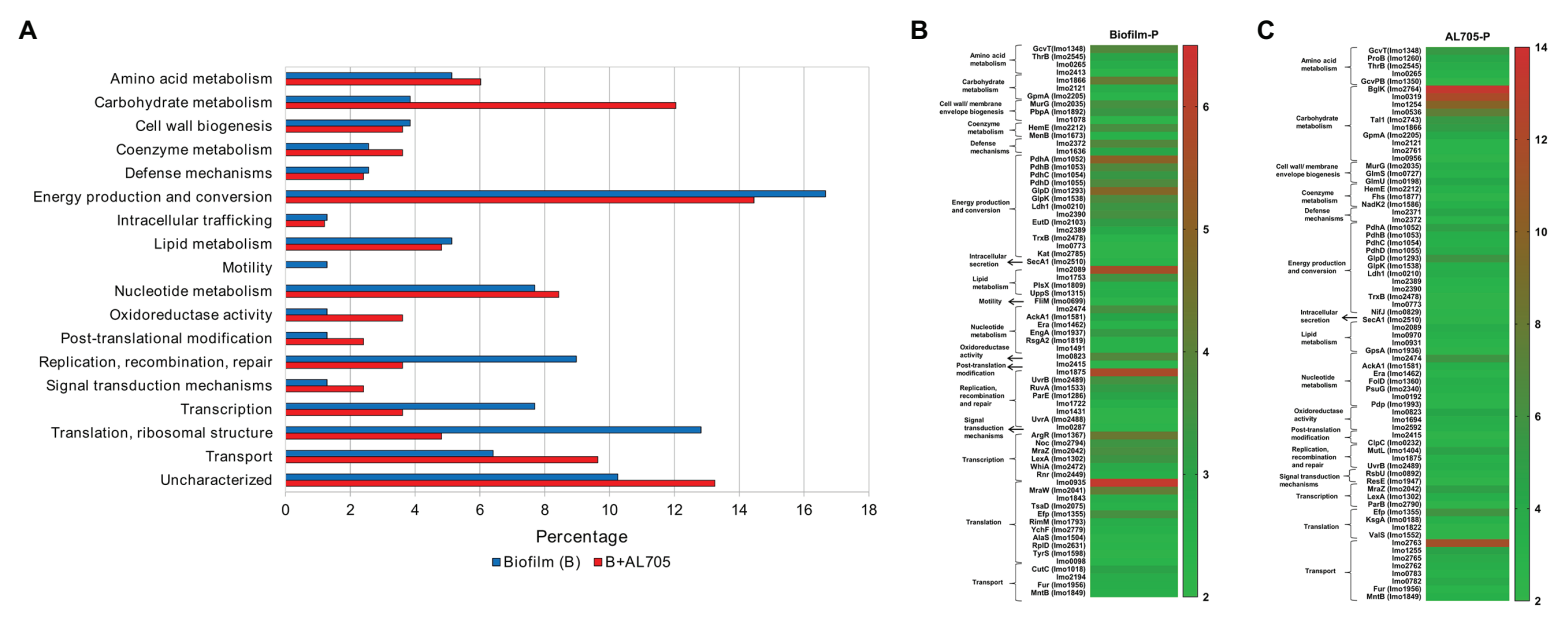
Up-regulated proteins by *L. monocytogenes* FBUNT untreated and bacteriocin AL705-treated sessile cells compared to planktonic cells: Horizontal bar chart showing the functional categories and proteins percentages **(A)** and Heat-map illustrating the fold change of the statistically significant overexpressed proteins in untreated **(B)** and treated **(C)** biofilm.

When cells in biofilm were compared to those in planktonic growth at 10°C, 78 statistically significant proteins were differentially expressed (Figure 1 and Supplementary Table S1); these were mostly related to energy production (16.7%), translation (12.8%), transcription (7.7%) and replication (9%) processes, transport (6.4%), nucleotide (7.7%), lipid (5.1%), carbohydrate (3.8%) and amino acid (5.1%) metabolism, and cell wall biogenesis (3.8%) categories (Figure 2A). In the pool of proteins regulated by *L. monocytogenes* FBUNT sessile cells, those whose expression was increased over 3.5-fold change (FC) were proteins involved in translation, lipid metabolism, and energy production (Figure 2B). Among proteins related to translation, putative tRNA (cytidine (34)-2'-O)-methyltransferase (Lmo0935; FC: 6.19) and ribosomal RNA small subunit methyltransferase H (MraW; FC: 4.05) as members of a superfamily implicated in tRNA processing, elongation factor P (Efp; FC: 3.53) as well as nucleotide-binding protein (Lmo2474; FC: 3.51) displaying ATPase/GTPase activity, were highly up-regulated. Likewise, the ability to modulate membrane fatty acid composition and improve membrane fluidity is a critical step for stress adaptation, thus lipase (Lmo2089; FC: 5.58) exhibiting esterase activity and lipid kinase (Lmo1753; FC 3.59) were among the more overexpressed proteins from lipid metabolism. Other proteins implicated in glycerol uptake and metabolism regulation, cell wall formation, and peptidoglycan biosynthesis were also up-regulated by FBUNT sessile cells at 10°C (Figure 2B). In addition, the overexpression of 13 proteins involved in energy production and conversion were detected, among them glycerol-3-phosphate dehydrogenase (GlpD; FC: 4.93) and glycerol kinase (GlpK; 3.53) involved in aerobic uptake of glycerol and the pyruvate dehydrogenase complex; E1 α subunit (PdhA; FC: 4.97), E1 β subunit (PdhB; FC: 3.55), dihydrolipoamide acetyltransferase (PdhC; FC: 3.28), dihydrolipoyl dehydrogenase (PdhD; FC: 3.57), and putative pyruvate phosphate dikinase regulatory protein (Lmo1866; FC: 3.8) were the most affected from carbohydrate metabolism. Regarding amino acid metabolism, protein aminomethyltransferase (GcvT; FC: 3.56), with a role in the pyruvate complex metabolism, was the most abundantly induced. On the other hand, although six proteins associated with transcription functionality were up-regulated, arginine repressor (ArgR; FC: 4.27) involved regulation of ADI (Arginine deiminase) pathway and transcriptional regulator (MraZ; FC: 3.57) were the most increased. In addition, ABC transporter ATP-binding proteins (Lmo1875 and Lmo2372; FC: 5.60 and 3.79) with nutrients transport function and oxidoreductase G (Lmo0823; FC: 3.78) from oxidoreduction function were up-regulated. A lower affected (FC < 3) protein, but with relevant function, was flagellar motor switch (FliM) related to cell motility and also induced by *L. monocytogenes* FBUNT during biofilm growth at 10°C.

### Up-Regulated Proteins by *L. monocytogenes* FBUNT Sessile Cells Treated With Lactocin AL705

When compared to planktonic growth, bacteriocin-treated sessile *L. monocytogenes* cells showed 83 statistically different expressed proteins (Figure 1 and Supplementary Table S1) mostly belonging to energy production (14.4%), carbohydrate metabolism (12%), transport (9.6%), and nucleotide metabolism (8.4%), as shown in Figure 2A. The up-regulation of 22 proteins with higher differential expression (FC ≥ 4) was observed in biofilm cells treated with the bacteriocin lactocin AL705 (Figure 2C). Among them, proteins from carbohydrate metabolism beta-glucoside kinase (BglK) that catalyze ATP-dependent phosphorylation of cellobiose, phospho-beta-glucosidase (Lmo0319), were involved in non-phosphorylated carbohydrates degradation, alpha, alpha-phosphotrehalase (Lmo1254) essential in disaccharide degradation, 6-phospho-beta-glucosidase (Lmo0536) with hydrolytic activity on phosphorylated β-glycosidic linkages and probable transaldolase 1 (Tal1) involving in pentose-phosphate pathway metabolites balance showed the highest overexpression (FC:13.43, 11.51, 9.79, 6.51, and 5.38, respectively). In addition, other proteins with lower expression, such as putative pyruvate phosphate dikinase regulatory protein (Lmo1866; FC: 4.97) and GpmA (FC: 3.97), were also regulated by lactocin AL705-treated sessile cells. Eight that induced proteins nutrients’ transport function were regulated in the presence of lactocin AL705; PTS cellobiose-specific IIC component (Lmo2763; FC: 11.12), PTS trehalose transporter subunit IIBC (Lmo1255; FC: 4.58), and ABC transporter permease (Lmo2371; FC: 4.25) were implicated in carbohydrate transmembrane transport and are integral membrane components that showed the highest overexpression, however other proteins were also increased. Similarly, amino acid metabolism represented by the overexpression of GcvT (FC: 5.38) and glutamate 5-kinase (ProB; FC: 4.15), which catalyze glycine degradation and phosphate transference to glutamate forming L-glutamate 5-phosphate, respectively, were the highest regulated, as well as glycerol-3-phosphate dehydrogenase (GlpD; FC: 5.71), PdhA (FC: 4.79), PdhB (FC: 3.38), PdhC (FC: 2.86), and PdhD (FC: 3.87), from energy production. Moreover, transcriptional regulator MraZ (FC: 4.78) and elongation factor P (Efp; FC: 5.87) proteins belonging to transcription/translation functionality were overexpressed, while Lmo2474 (FC: 5.64) and Lmo0823 (FC: 4.16) from nucleotide metabolism and oxidoreduction function were also up-regulated when lactocin AL705 was added to *L. monocytogenes* FBUNT biofilm.

### Differentially Regulated Proteins Comparing *L. monocytogenes* FBUNT Lactocin AL705-Treated and Untreated Sessile Cells

**Figure 3:**
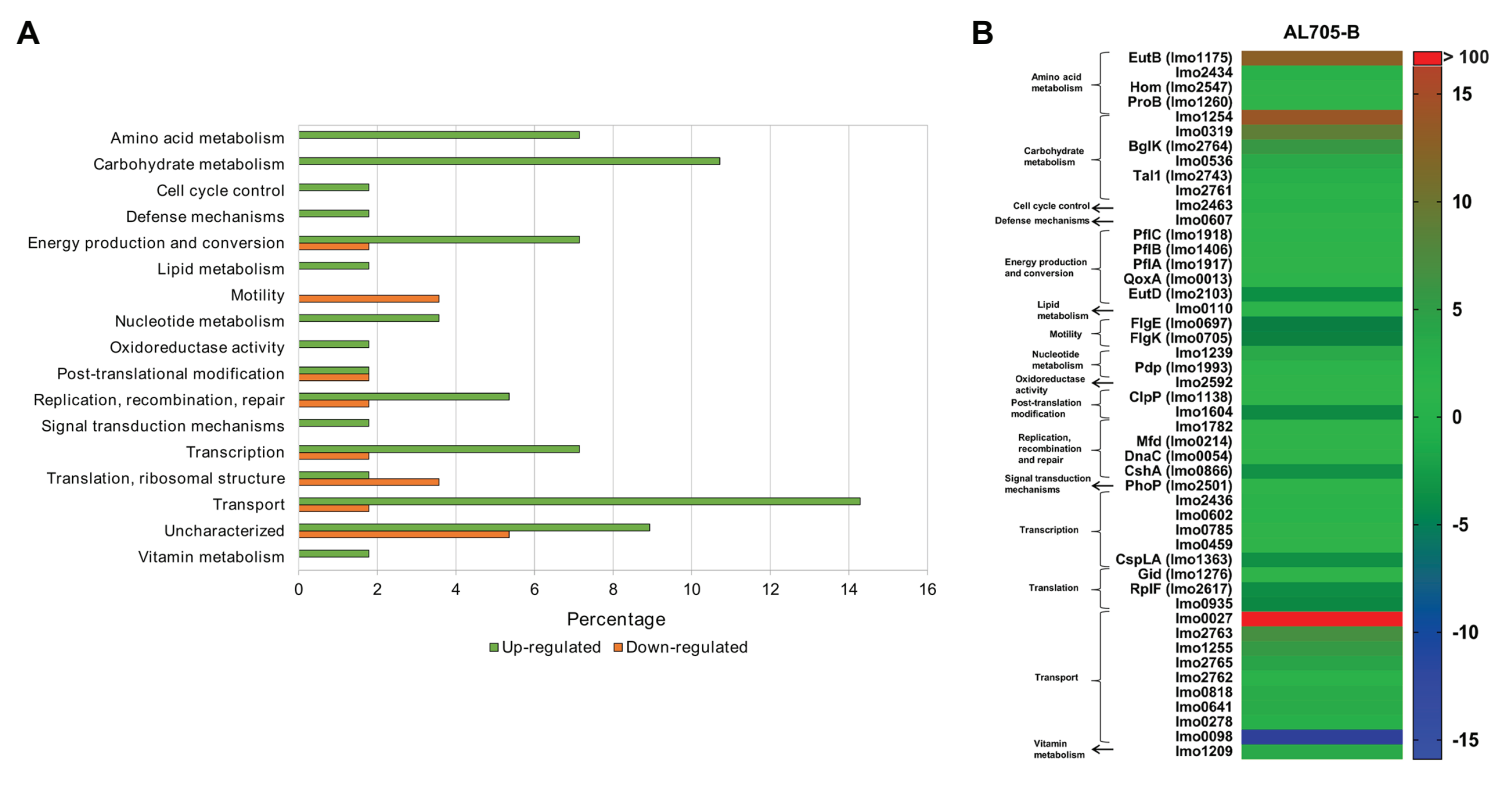
Regulated proteins by *L. monocytogenes* FBUNT sessile cells treated with bacteriocin AL705 compared to untreated biofilm cells: Horizontal bar chart showing the functional categories and percentages of over and downexpressed proteins **(A)** and Heat-map illustrating the fold change of the differentially expressed proteins in treated biofilm **(B)**.

Bacteriocin-treated sessile cells showed 56 differentially expressed proteins when compared to untreated sessile cells at 10°C (Supplementary Table S2). Among overexpressed proteins (FC > 4), most of them belonged to transport (16%), carbohydrate (10.7%), amino acid (7.1%), and nucleotide metabolism (3.6%) functional categories (Figure 3A). As shown in Figure 3B, proteins with nutrients’ transport function were highly up-regulated by bacteriocin-treated sessile cells when compared to untreated biofilm. PTS beta-glucoside transporter subunit IIABC (Lmo0027; FC: 123.9) showed a remarkably high expression, whereas other proteins, such as PTS cellobiose transporter subunit IIC (Lmo2763), subunit IIB (Lmo2762), and subunit IIA (Lmo2765), PTS trehalose transporter subunit IIBC (Lmo1255) that catalyzes phosphorylation and translocation across the cell membrane (FC: 7.50, 3.31, 5.26, and 6.49), cation-transporting ATPase (Lmo0818; FC: 4.46), and heavy metal-transporting ATPase (Lmo0641; FC: 4.45), which enable cation and solutes transmembrane transport, were also up-regulated. Three of these proteins (Lmo2763, Lmo1255, and Lmo2765) shared overexpression when compared to the planktonic condition. Nevertheless, PTS mannose transporter subunit IID (Lmo0098), that was regulated during biofilm formation, was strongly decreased (FC: −14.78) in the presence of the bacteriocin. On the other hand, among six differentially induced proteins from carbohydrate metabolism, Lmo1254 (FC: 14.28), Lmo0319 (FC: 9.62), BglK (FC: 6.84), amd Lmo0536 (FC: 4.62) from glycolysis pathway participating in oligosaccharides degradation/phosphorylation processes were up-regulated by bacteriocin-treated sessile cells, with alpha, alpha-phosphotrehalase (Lmo1254) protein showing the maximum overexpression. These six proteins from carbohydrate metabolism up-regulated by lactocin AL705-treated sessile cells were the same regardless of the comparison (planktonic or untreated biofilm). Moreover, *Listeria* sessile cells greatly overexpressed the protein EutB (FC: 13.09) with ethanolamine ammonia-lyase activity involved in the catabolic process of amino acids, and dITP/XTP pyrophosphatase (Lmo1239; FC: 4.48) from nucleotide metabolism catalyzing nucleoside triphosphates to monophosphate hydrolysis acts as a house-cleaning enzyme avoiding chromosomal lesions. Concerning the transcription/translation function, several proteins regulated their abundance with FC < 2.2.

### Proteins Expressed Exclusively by *L. monocytogenes* FBUNT Biofilm Treated With Lactocin AL705

As stated above, some proteins expressed in sessile cells treated with bacteriocin were unable to be compared with planktonic and untreated biofilm because they were absent in both conditions (Table 1). Among them, 44 proteins mainly related to amino acid (ethanolamine), carbohydrate (1,2-propanediol), lipid (cardiolipin synthase and *dltA*), nucleic acid, vitamin metabolism, transcription/translation (*hrcA*), and transport were overexpressed.

**Table 1:** Proteins identified exclusively in biofilm cells treated with lactocin AL705.

Categories	Protein name	Locus/gene	N° Accession
Amino acid metabolism	Ethanolamine ammonia-lyase small subunit	*lmo1176/eutC*	Q8Y7U4
	Carboxysome structural protein	*lmo1177/ eutL*	Q8Y7U3
	Ethanolamine utilization protein	*lmo1187/eutQ*	Q8Y7T4
Carbohydrate metabolism	Aldose 1-epimerase	*lmo2476*	Q8Y4G7
	Gluconate kinase	*lmo2712*	Q8Y3W7
	Propanediol dehydratase subunit alpha	*lmo1153*	Q8Y7W6
	Pyruvate, phosphate dikinase	*lmo1867*	Q8Y633
	Sugar kinase	*lmo2341*	Q8Y4U1
	Transketolase	*lmo0342*	Q8YA23
Coenzyme metabolism	Molybdenum cofactor biosynthesis protein B	*lmo1048*	Q8Y869
Defense mechanisms	ABC transporter ATP-binding protein	*lmo1131*	Q8Y7Y8
	ABC transporter ATP-binding protein	*lmo2215*	Q8Y561
Energy production and conversion	L-lactate dehydrogenase	*lmo1057*	Q8Y860
	NADPH-dependent butanol dehydrogenase	*lmo1171/pduQ*	Q8Y7U8
	Formate dehydrogenase subunit alpha	*lmo2586*	Q8Y469
Lipid metabolism	Cardiolipin synthase	*lmo0008*	Q8YAV5
	D-alanine-poly(phosphoribitol) ligase subunit 1	*lmo0974/dltA*	Q8Y8D4
Post-translational modification, protein turnover, and chaperones	Protease HtpX homolog	*lmo0963/htpX*	Q8Y8E1
Replication, recombination, and repair	ATP-dependent dsDNA exonuclease SbcC	*lmo1645*	Q8Y6N9
	Excinuclease ABC subunit A	*lmo2050*	Q8Y5K9
Signal transduction mechanisms	Response regulator	*lmo1507*	Q8Y719
	Sensor histidine kinase	*lmo1021*	Q8Y893
Transcription	Transcriptional regulator	*lmo0776*	Q8Y8W6
	DeoR family transcriptional regulator	*lmo2366*	Q928R7
	LacI family transcriptional regulator	*lmo2128*	Q8Y5D7
	Heat-inducible transcription repressor HrcA	*lmo1475/hrcA*	P0DJM4
Transport of carbohydrate and inorganic ion	PTS mannose transporter subunit IIB	*lmo0784*	Q8Y8V8
	PTS mannose transporter subunit IID	*lmo2000*	Q8Y5Q6
	PTS mannose transporter subunit IIB	*lmo2002*	Q8Y5Q4
	Ferrichrome-binding protein	*lmo1959*	Q8Y5U6
	Heavy metal-transporting ATPase	*lmo1853*	Q8Y647
Vitamin metabolism	Demethylmenaquinone methyltransferase	*lmo1931/ubiE*	P67055
	Dihydropteroate synthase	*lmo0224/sul*	Q8YAC2
	1,4-dihydroxy-2-naphthoate octaprenyltransferase	*mo1677/menA*	Q8Y6K7
	Precorrin-3 methylase	*lmo1197/cbiF*	Q8Y7S4
Uncharacterized	Hypothetical protein	*lmo2209*	Q8Y567
	Hypothetical protein	*lmo2486*	Q8Y4F8
	Hypothetical protein	*lmo1466*	Q8Y746
	Hypothetical protein	*lmo2843*	Q8Y3J2
	Hypothetical protein	*lmo0391*	Q8Y9X6
	Hypothetical protein	*lmo0111*	Q8YAK7
	Hypothetical protein	*lmo2502*	Q8Y4E4
	Hypothetical protein	*lmo0584*	Q8Y9E6
	Hypothetical protein	*lmo1452*	P53434

### Interaction of Differentially Regulated Proteins in *L. monocytogenes* FBUNT Untreated and Treated Biofilm Cells

**Figure 4:**
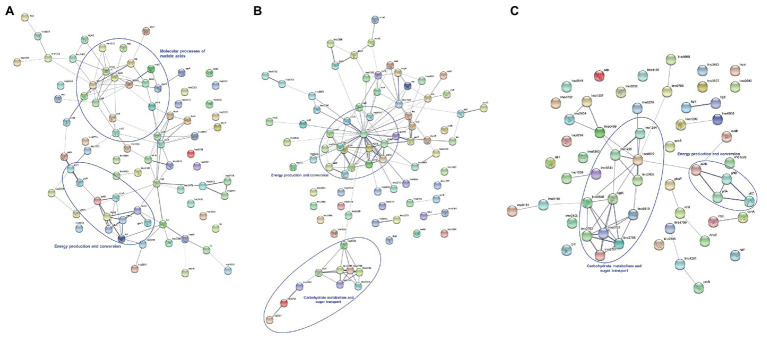
Protein-protein interactions of differentially expressed proteins identified by comparison of untreated biofilm **(A)**, lactocin 705-treated biofilm **(B)** with planktonic cells and between sessile cells with and without treatment **(C)**. The thickness of lines between the nodes indicating the degree of interaction.

The protein-protein interaction networks were constructed analyzing proteins that showed differences in their abundance in the following three comparisons: untreated and treated sessile cells regarding to the planktonic state (Figures 4A,B) and untreated vs. lactocin-treated biofilm cells (Figure 4C). As shown in Figure 4A, 17 out of the 78 proteins up-regulated in biofilm with respect to the planktonic cells have no interactions with each other. However, 61 proteins are related in the network. Most proteins with strong interaction are related with energy production and conversion (PdhABCD, EutD, and Ldh) whereas the remaining are mainly involved in nucleic acids molecular processes such as transcription, translation, replication, recombination, and repair (RplD, EngA, Lmo1843, Era, YchF, TsaD, Efp, MraW, and MraZ). In the network of treated sessile cells (Figure 4B), 64 out of the 83 proteins showed interaction among them; the strongest associations were also for proteins related with energy production and conversion (PdhABCD, NifJ, Ldh, GlpD, and GlpK) as well as among proteins involved in carbohydrate metabolism and sugar transport (Lmo2761, Lmo2762, Lmo2763, Lmo2765, Lmo1255, Lmo1254, Lmo2121, Lmo0319, Lmo0536, and BglK). The third network (Figure 4C) corresponds to the protein-protein interactions of the comparison between untreated and lactocin-treated sessile cells, in which 24 proteins are associated. The majority of the proteins showing strong interactions are those related to carbohydrate metabolism and sugar transport (Lmo2761, Lmo2762, Lmo2763, Lmo2765, Lmo0536, BglK, Lmo0319, Lmo2436, Lmo1255, Lmo1254, and Lmo0027), and with many of these protein associations the same result was found in the comparison with planktonic cells. Also, high interaction among PflABC and EutD proteins from energy production was observed.

## Discussion

### Quantitative Proteomic of *L. monocytogenes* FBUNT Growing in Biofilm at 10°C

Bacteria have the ability to adapt to the surrounding environment; adaption mechanisms allowed *L. monocytogenes* to survive hostile conditions, such as low temperature (Durack et al., 2013; Santos et al., 2019), while the presence of antimicrobial compounds have been recently addressed (Caballero Gómez et al., 2013; Liu et al., 2014; Lanciotti et al., 2019). An understanding of the effects of natural antimicrobials on the bacterial cell physiology and virulence is necessary for their exploitation in the food industry as alternatives to traditional preservatives. Particularly, biofilm is the lifestyle of choice for *L. monocytogenes*, representing a stable condition that provides nutrient resources and protection against harsh environments (Gandhi and Chikindas, 2007). Comparative proteomic analyses of *L. monocytogenes* FBUNT during biofilm formation at 10°C in the presence or absence of the bacteriocin lactocin AL705 showed different relative abundances of regulated proteins.

Compared to planktonic cells at 10°C, major protein shifts by *L. monocytogenes* FBUNT growing in biofilm were those related to transcription/translation (Lmo0935; MraW; MraZ; ArgR) and energy conversion and production (GlpD, PdhABCD, and Ldh1). Lmo0935 and MraW proteins from the superfamily involved in tRNA processing play a role in fine-tuning the shape and function of the peptidyl (P)-site in the ribosome, thus increasing decoding fidelity and avoiding stalling in translation. In addition, the DNA-binding transcriptional regulators MraZ and EngA proteins play an essential role in the late steps of ribosome biogenesis; response of *L. monocytogenes* to stress factors appears to involve activation of ribosomal gene transcription (Durack et al., 2013). On the other hand, ArgR protein overexpression was reported as a response to various environmental stimuli, such as changes in arginine and other metabolites concentration, pH, oxygen tension, and temperature variations (Ryan et al., 2009; Cheng et al., 2017). Also, in order to adapt to stressful environments, bacteria must be able to repair damaged DNA and synthesize mRNA and proteins. In this study, UvrB, LexA, RuvA, and Kat proteins related to DNA damage recognition and repair were up-regulated in coincidence to that described for strain EGD-e biofilm formation in which a high number of proteins associated with repair system mechanisms were induced at low temperatures (Santos et al., 2019). Particularly, RuvA and LexA proteins were overexpressed as cross protection provided by cold and osmotic stress by EGD-e strain (Pittman et al., 2014), while Kat protein was induced during cold and oxidative stress (Soni et al., 2011). On the contrary, UvrB and Kat proteins were downregulated under redox shock indicating that this stress seems to prevent DNA damage (Ignatova et al., 2013). Moreover, in order to maintain membrane fluidity and functionality, bacteria modify their physical properties by changing the fatty acid composition, this being in coincidence with the lipid biosynthesis activation by *L. monocytogenes* FBUNT sessile cells. Indeed, the overexpression of Lmo2089 and Lmo1753 proteins agree with the regulated proteins from lipid metabolism by other *Listeria* strains during growth at low temperatures (Chan et al., 2007) and *Lb. sakei* during biofilm formation on stainless steel at 10°C (Pérez-Ibarreche et al., 2017).

Protein expression related to energy production and conversion were highly affected by *L. monocytogenes* FBUNT growing in biofilm at 10°C. When compared to planktonic cells, the PdhABCD complex and Ldh1 proteins involved in cellular metabolism and energy production, specifically the transformation of pyruvate to acetyl-CoA and to lactate, were up-regulated, this being in agreement with recently reported results for *L. monocytogenes* EGD-e in biofilm formed at low temperature (Santos et al., 2019). In addition, high induction of PdhA and PdhB proteins were shown in redox shock (Ignatova et al., 2013); a downregulation occurred under heat shock conditions (Ágoston et al., 2009). Given that TSB-YE used in this study is a complex medium, carbohydrates other than glucose are available to be transported into the cell; metabolism of alternative carbohydrates is advantageous during stress conditions (Pittman et al., 2014). Even moderately, regulation of Lmo1866 and maltose phosphorylase (Lmo2121) was increased by *L. monocytogenes* FBUNT sessile cells. The induction of Efp and HemE proteins have been shown to have chaperone-like functions by aiding in proper protein folding proteins (Caldas et al., 2000). Up-regulation of these proteins were reported for different *L. monocytogenes* strains under stress conditions (Cacace et al., 2010; Ignatova et al., 2013; Pittman et al., 2014).

Moreover, the overexpression of oxidoreductase (Lmo0823) protein was in accordance with its induction during biofilm growth of EGD-e strain at 10°C (Santos et al., 2019). Exposure to low temperatures was reported to enhance oxidative stress; indeed, higher levels of superoxide dismutase, catalase, and proteins similar to oxidoreductase aldo/keto reductase family were regulated by *L. monocytogenes* during cold adaptation (Cacace et al., 2010). In addition, *L. monocytogenes* FBUNT sessile cells growing at 10°C showed a low induction of FliM protein compared to planktonic cells. It is known that FliM, FlhB, and FliY proteins constitute the flagellar basal structure required for flagellum synthesis in *L. monocytogenes* EGD-e (Cheng et al., 2018). Flagella motility is a highly advantageous but energetically demanding survival mechanism used by bacteria in extracellular environments. It was reported that *L. monocytogenes* flagella are used for motility, not as adhesins to increase host cell invasion (O’Neil and Marquis, 2006), thus it is non-critical for biofilm formation on surfaces, since disruptions in flagella-related genes resulted in immotile cells increasing biofilm formation (Piercey et al., 2016).

### Quantitative Proteomic of *L. monocytogenes* FBUNT Biofilm Treated With Lactocin AL705 at 10°C

The obtained results showed differential protein expression depending on FBUNT cellular state (planktonic or sessile) as well as biofilm formation in the presence or absence of lactocin AL705. Compared with planktonic growth in the absence of lactocin, *L. monocytogenes* sessile cells treated with a sublethal dose of the bacteriocin overexpressed proteins mostly implicated in carbohydrate metabolism and nutrient transport, this indicating their function to enhance energy production (Figure 2C). Indeed, the proteins BglK, Tal1, Lmo0536, Lmo0319, and Lmo1254 were involved in the intracellular catabolic processes of cellobiose and trehalose to glucose were among the highest regulated; however, the expression of Lmo1866 and GpmA (2, 3-bisphosphoglycerate-dependent phosphoglycerate mutase) proteins were also increased even in a lower abundance. Carbohydrates play important roles in the adaptive response of bacteria in a stress environment. It seems that overexpression of carbohydrates’ catabolism proteins by sessile cells could be an attempt to compensate for impaired energy generation caused by sub-inhibitory concentrations of lactocin AL705 interacting with the cytoplasmic membrane. The induction of proteins from carbohydrate metabolism and energy production is in agreement with that reported for *L. monocytogenes* EGD-e in biofilm at 10°C (Santos et al., 2019). On the other hand, several transport-associated proteins were regulated with high FC in the biofilm + lactocin AL705 condition. *L. monocytogenes* possesses a large number of transport systems for sugars and sugar derivatives. Ion-driven transporters, ATP binding cassette (ABC) transporters, and the phosphoenolpyruvate carbohydrate phosphotransferase system (PTS) require energy for the carbohydrate uptake reaction, sugars-phosphates being taken up *via* ion-driven permeases (Deutscher et al., 2014). In the presence of lactocin AL705, two ABC transporters of nutrients across the cell membrane were regulated with low abundance changes, however, a high regulation of cellobiose IIC-PTS and trehalose IIBC-PTS (Lmo2763 and Lmo1255) and a minor increase of mannose IIB/IIC/IID-PTS (Lmo0783, Lmo0782, Lmo0098) proteins were observed upon the addition of the bacteriocin during *L. monocytogenes* FBUNT biofilm formation. Most carbohydrates (glucose, fructose, mannose, cellobiose, and trehalose, among others) used by *L. monocytogenes* are transported into the cell by PTS. In bacteria, the PTS system in general, and the mannose PTS in particular, have a central role in a range of regulatory events controlling carbon metabolism. In addition, even when these changes in metabolism may affect bacterial cell wall and cell membrane composition, regulation of proteins from lipid metabolism by sessile cells in the presence of lactocin AL705 showed low changes in their abundance compared to the destabilized cell envelope state of sakacin P resistant-*L. monocytogenes* mutants (Tessema et al., 2009). It was reported that certain carbohydrates taken up *via* PTS strongly repress the expression of virulence genes (Deutscher et al., 2014). Indeed, the hydrolysis of easily metabolized sugars are repressed in the presence of glucose, thus, these carbohydrates (mainly cellobiose) in the media strongly affected hemolysin and PrfA transcription by *L. monocytogenes*. The presence of cellobiose and other PTS sugars in the environment might indicate that the saprophytic lifestyle is favored, so there was no need to express virulence genes (Gilbreth et al., 2004; Deutscher et al., 2014). In this study, the bacteriocin addition to *L. monocytogenes* FBUNT biofilm at 10°C seemed to induce an active carbohydrate metabolism, which would prevent expression of virulence genes. Pyruvate can be shunted to acetate, acetoin, and lactate or converted to acetyl-CoA by pyruvate dehydrogenase complex (Pdh) or pyruvate formate lyase (Pfl) (Sauer et al., 2019). The PdhABCD complex was up-regulated in comparison to planktonic state while PflABC proteins were overexpressed regarding untreated biofilm. It has recently been demonstrated that pyruvate is required for the biofilm formation of other bacterial species, such as *Pseudomonas aeruginosa*, and that the depletion of pyruvate results in biofilm dispersion and increases the antibiotic susceptibility (Goodwine et al., 2019). Thus, a possible hypothesis could be that a similar behavior is occurring in *L. monocytogenes* in response to lactocin.

Moreover, the up-regulation of GcvT protein involved in amino acid metabolism and transport is in coincidence with that reported for *L. monocytogenes* EGD-e biofilm at 10°C (Santos et al., 2019). In particular, the overexpression of ProB as part of the proline biosynthesis in sessile cells upon lactocin AL705 addition may lead to intracellular proline accumulation acting as a compatible solute promoting pathogen adaption to salt and chill conditions (Sleator et al., 2003). Overexpressed Lmo2474 and Lmo0823 proteins implicated in nucleotides metabolism and oxidoreduction processes, respectively, would suggest the need for sessile *L. monocytogenes* cells treated with the bacteriocin at 10°C to remain viable through DNA damage prevention. In addition, FBUNT strain seemed to share the same proteins (MraZ and Efp) involved in transcription/translation both in biofilm and biofilm + lactocin AL705 compared with planktonic condition. From these results, it can be highlighted that the addition of lactocin AL705 to *L. monocytogenes* sessile cells at 10°C triggered important changes with the major regulation of glycolysis and PTS proteins.

On the other hand, bacteriocin addition to sessile cells when compared to untreated biofilm caused the highest changes in proteins’ differential expression (Figures 1, 3B and Supplementary Table S2). Major up-regulation was found for proteins involved in transport as well as carbohydrate and amino acid metabolism. The overexpression of transport proteins (PTS-cellobiose IIC/IIA and PTS-trehalose IIBC) were common regardless of the comparison (planktonic or biofilm), however, PTS beta-glucoside transporter subunit IIABC (Lmo0027) and PTS mannose transporter subunit IID (Lmo0098) proteins resulted the most increased and decreased respectively, while the Lmo0027 protein was only regulated by *L. monocytogenes* FBUNT sessile cells when compared with untreated biofilm. It is known that most carbohydrates used by *L. monocytogenes* are transported by PTS, among them the disaccharides cellobiose, trehalose, and mannose. The high expression of Lmo0027 protein (PTS glucose permease) in this study is related to the saprophytic growth of *L. monocytogenes*, the presence of environment-specific signals such as cold, acid, and nutrient stress conditions being responsible for triggering the up-regulation of this permease (Stoll and Goebel, 2010). In addition, the α-disaccharide trehalose was reported to be taken up *via* a PTS transporter (Lmo1255), which has been shown to complement with the overexpressed enzyme α, α-(1,1) phosphotrehalase (Lmo1254) involved in the hydrolysis of trehalose-6-P into glucose and glucose-6-P. Up-regulation of this protein by *L. monocytogenes* FBUNT prevented trehalose accumulation in the cytosol that would decrease stress resistance of the bacterium (Ells and Truelstrup Hansen, 2011; Deutscher et al., 2014). However, the action of lactocin AL705 on FBUNT sessile cells showed a strong downregulation of mannose IID-PTS (Lmo0098) agreeing with the role of this protein as a target of class IIa bacteriocins (Tessema et al., 2009). *L. monocytogenes* was reported to limit the expression of PTS proteins to minimize the production of targets to class II bacteriocins (Diep et al., 2007; Liu et al., 2014). The membrane protein IID and IIC of the mannose-PTS form a membrane-located complex, serving as a receptor for class IIa bacteriocins (Kjos et al., 2011; Deutscher et al., 2014). Indeed, a role of mannose-PTS target downregulation may be assigned during *L. monocytogenes* FBUNT adaption to sub-inhibitory lactocin AL705 concentrations. Besides PTS transporters, even with low abundance changes, inorganic ions (Lmo0818 and Lmo0641) and sugar ABC (Lmo0278) transport associated proteins, as well as a protein (Lmo0607) involved in defense mechanisms, were also up-regulated by *L. monocytogenes* FBUNT sessile cells compared with untreated biofilm. These results suggest that the addition of lactocin AL705 to biofilm cells imposed greater carbohydrate metabolism changes compared to planktonic and biofilm cellular states; cells exhibit an enhanced demand of energy to sustain growth. Recent studies reported that the ABC transporters are related to resistance against antimicrobials, especially those that act at a cell membrane level (Grubaugh et al., 2018). In addition, other proteins imvolved in inorganic ions transport were up-regulated, their abundance being also reported for *L. monocytogenes* EGD-e sessile cells at low temperatures (Durack et al., 2013; Santos et al., 2019). The regulation of these transporters may be related to the cell effort to counteract the effect of the antimicrobial. Likewise, up-regulation of the house-cleaning enzyme Lmo1239 involved in the modulation of nucleotides’ structure agrees with those previously reported for *L. monocytogenes* subjected to osmotic and cold stress (Durack et al., 2013; Pittman et al., 2014). A downregulation of CspLA (Lmo1364) upon lactocin AL705 treatment was observed; on the contrary, the expression of other cold shock proteins were reported to be enhanced by *L. monocytogenes* LO28 exposed to low temperature and high hydrostatic pressure (Wemekamp-Kamphuis et al., 2002). The difference may be due to cell physiological state (biofilm) compared to other studies performed with planktonic cells of different strains (Wemekamp-Kamphuis et al., 2002; Durack et al., 2013). In addition, the repression of the cold shock protein CspLA in this study could be related to a lack of adaption of FBUNT strain to environmental stress caused by the presence of the bacteriocin.

Moreover, the up-regulation of EutB protein with ethanolamine ammonia-lyase activity by sessile cells treated with lactocin AL705 compared to untreated biofilm has been shown (Figure 3B). This protein is derived from the membrane phospholipid phosphatidylethanolamine and is a prevalent compound in the gastrointestinal tract and in foods moderately rich in fat that can be used as a carbon, nitrogen, and/or energy source. A number of host environments have activated the expression of *eut* gene in *L. monocytogenes*; an appreciation for how ethanolamine (EA) contributes to infection and colonization in host and foods has emerged (Tang et al., 2015; Kaval and Garsin, 2018). *L. monocytogenes* has been revealed to codify for all necessary enzymes for the utilization of EA as well as 1,2-propanediol, which is produced during rhamnose and fucose catabolism (Orsi et al., 2015). A close relationship between EA utilization and cobalamin biosynthesis/transport pathways was also demonstrated in *L. monocytogenes* (Mellin et al., 2013; Tang et al., 2015). The ability to synthetize vitamin B_12_ and to use EA and 1,2-propanediol give this pathogen a selective advantage against other microorganisms during food contamination and host infection. Interestingly, a lack of regulation and downregulation of motility-associated proteins were observed upon addition of lactocin AL705 to *L. monocytogenes* FBUNT sessile cells, when compared to planktonic and untreated biofilm conditions, respectively. As stated above, the lack of flagella proteins was associated with enhanced biofilm formation under static conditions, thus the bacteriocin addition had no impact on motility protein regulation, however pore formation by class IIa lactocin AL705 may destabilize the bacterial cell membrane, causing alteration in motility.

### Proteins Expressed Exclusively by *L. monocytogenes* FBUNT Biofilm Treated With Lactocin AL705

A number of proteins belonging to different functional categories were uniquely expressed in sessile cells growing at 10°C treated with the bacteriocin. Among them, three proteins (EutC, Lmo1176; EutL, Lmo1177; EutQ, Lmo1187) involved in the metabolic process of EA were regulated. As previously described, EA as a valuable source of carbon and/or nitrogen can be catabolized by *L. monocytogenes* through a complex set of genes (Orsi et al., 2015). The previously regulated EutB protein, together with EutC, constitute the two subunits of the Ethanolamine ammonia lyase implicated in the degradation of acetaldehyde and ammonia; a range of accessory genes that enhance EA breakdown were found together in a strain-dependent complex operon (Kaval and Garsin, 2018). In addition to EutL that has structural activity, EutQ as an acetate kinase is required for EA catabolism under certain conditions, its degradation being enhanced when the protein is localized in the bacterial microcompartments, the polyhedral organelles in which related enzymes and auxiliary proteins are encapsulated within a shell (Kerfeld et al., 2010). In addition, the coenzyme-B_12_-dependent 1,2-propanediol produced by anaerobic degradation of fucose and rhamnose sugars is used as a carbon and energy source by *L. monocytogenes* growing on cold smoking salmon (Tang et al., 2015). Accordingly, proteins related to 1,2-propanediol utilization (*pdu* operon) were up-regulated by sessile cells of FBUNT strain such as Lmo1153 (hydrolyase activity-cobalamin binding) and PduQ (oxidoreductase activity). In *L. monocytogenes*, cobalamin-binding riboswitches located upstream of the *eut* and *pdu* locus regulate the expression of EA and 1,2-propanediol, demonstrating the close relationship between EA and 1,2-propanediol utilization pathways and cobalamin biosynthesis/transport pathways (Mellin et al., 2013). In addition, sessile cells of *L. monocytogenes* FBUNT up-regulated MenG and MenA proteins as part of the menaquinone (Vitamin K2) biosynthesis pathway, and Sul protein was implicated in the tetrahydrofolate biosynthesis as a response to cold and lactocin AL705. The ability to synthetize vitamin B_12_ and other vitamins together with the utilization of EA and 1,2-propanediol would facilitate *L. monocytogenes* FBUNT adaption and survival by increasing nutrient sources to maintain its viability. Proteins Lmo0008 and DltA (Lmo0974) implicated in membrane phospholipids metabolism and lipoteichoic acid biosynthesis and those related to ABC transport ATP-binding and mannose-PTS membrane-located complex, reported to serve as receptors of class IIa bacteriocins, were additionally expressed by treated sessile cells. Interestingly, the heat-inducible transcription repressor HrcA (Lmo1475) was also expressed by *L. monocytogenes* FBUNT biofilm cells treated with lactocin AL705. This protein is a negative regulator preventing induction of heat shock genes (*grpE*, *dnaK*, *dnaJ*, *groEL*, and *groES*) which, as expected, were not identified in this study due to the growth conditions (10°C).

## Conclusion

*L. monocytogenes* is widespread in the natural environment as saprophyte and is able to overcome various kinds of stress, including low temperatures and traditional sanitizers used in the food industry. Its ability to adapt and multiply in such conditions is partially due to its ability to form biofilm. In this study, the treatment of *L. monocytogenes* FBUNT sessile cells growing at 10°C with the bacteriocin lactocin AL705 at sub-inhibitory concentration resulted in the adaption of the pathogen through the overexpression of stability mechanisms by modulation of nucleic acids structures, structural integrity of cell membranes, carbohydrate and amino acids metabolism, and transport, however genes associated with motility were not critical for biofilm formation. Lactocin AL705 addition to biofilm cells seemed to induce an active carbohydrate catabolism as an attempt to compensate for impaired energy generation caused by the bacteriocin interaction with the membrane. Among catabolized carbohydrates taken up *via* multiple PTS transporters, cellobiose-PTS would indicate that the saprophytic lifestyle is favored, so there was no need to express virulence genes, while the downregulation of mannose-PTS is related to its role as a class IIa bacteriocins target. These suggest that the treatment of biofilm with lactocin AL705 imposed greater carbohydrate metabolism changes compared to planktonic and biofilm cellular states; cells exhibited an enhanced demand of energy to sustain growth to protect *L. monocytogenes* FBUNT from the action of the bacteriocin. The proteomic approach used in this study may aid in highlighting potential target genes/proteins of bacteriocins’ stress resistance that will be of particular interest for the development of *L. monocytogenes* control strategies in food processing.

## Data Availability Statement

The raw data supporting the conclusions of this article will be made available by the authors, without undue reservation.

## Author Contributions

CM performed laboratory experiments. LM designed the study. GV, PC, LM, and CM analyzed and discussed the data/results. FS designed the graphics. GV, CM, and LM wrote the manuscript. All authors contributed to the article and approved the submitted version.

### Conflict of Interest

The authors declare that the research was conducted in the absence of any commercial or financial relationships that could be construed as a potential conflict of interest.
