# Supplementary material for: Proteomic Analysis of Listeria monocytogenes FBUNT During Biofilm Formation at 10°C in Response to Lactocin AL705
Source: Front Microbiol. 2021 Jan 29;12:604126. doi: 10.3389/fmicb.2021.604126 (PMC7880126; doi:10.3389/fmicb.2021.604126)
Supplement: Supplementary file 1 [file Data_Sheet_1.docx]

Supplementary Material

**Table S1.** Differentially expressed proteins by *L. monocytogenes* FBUNT cells in untreated and treated with bacteriocin AL705 biofilm compared to planktonic cells.

| **Categories** | **Protein name** | **Locus/Gene** | **N° Accession** | **Treatment** | **Fold change** |
| --- | --- | --- | --- | --- | --- |
| **Amino acid metabolism** | Aminomethyltransferase | *lmo1348/gcvT* | Q8Y7D5 | Biofilm | 3.56 |
|  |  |  |  | B+ AL705 | 5.38 |
|  | Homoserine kinase | *lmo2545/thrB* | Q8Y4A6 | Biofilm | 2.93 |
|  |  |  |  | B+ AL705 | 2.20 |
|  | Succinyl-diaminopimelate desuccinylase | *lmo0265* | Q7AP85 | Biofilm | 2.57 |
|  |  |  |  | B+ AL705 | 2.02 |
|  | Cysteine desulfurase | *lmo2413* | Q8Y4M4 | Biofilm | 2.14 |
|  | Glutamate 5-kinase | *lmo1260/proB* | Q93Q56 | B+ AL705 | 4.15 |
|  | Probable glycine dehydrogenase subunit 2 | *lmo1350/gcvPB* | Q8Y7D3 | B+ AL705 | 2.43 |
| **Carbohydrate metabolism** | Putative pyruvate, phosphate dikinase regulatory protein | *lmo1866* | Q8Y634 | Biofilm | 3.80 |
|  |  |  |  | B+ AL705 | 4.97 |
|  | Maltose phosphorylase | *lmo2121* | Q8Y5E3 | Biofilm | 2.50 |
|  |  |  |  | B+ AL705 | 2.69 |
|  | 2,3-bisphosphoglycerate-dependent phosphoglycerate mutase | *lmo2205/gpmA* | Q8Y571 | Biofilm | 2.17 |
|  |  |  |  | B+ AL705 | 3.97 |
|  | Beta-glucoside kinase | *lmo2764/bglK* | Q8Y3R9 | B+ AL705 | 13.43 |
|  | Phospho-beta-glucosidase | *lmo0319* | Q7AP84 | B+ AL705 | 11.51 |
|  | Alpha,alpha-phosphotrehalase protein | *lmo1254* | Q8Y7M0 | B+ AL705 | 9.79 |
|  | 6-phospho-beta-glucosidase | *lmo0536* | Q92EC0 | B+ AL705 | 6.51 |
|  | Probable transaldolase 1 | *lmo2743/tal1* | Q8Y3T8 | B+ AL705 | 5.38 |
|  | Beta-glucosidase | *lmo2761* | Q8Y3S1 | B+ AL705 | 2.74 |
|  | N-acetylglucosamine-6-phosphate deacetylase | *lmo0956* | Q8Y8E8 | B+ AL705 | 2.22 |
| **Cell wall/membrane/envelope biogenesis** | UDP-diphospho-muramoylpentapeptide beta-N-acetylglucosaminyltransferase | *lmo2035/murG* | Q8Y5M2 | Biofilm | 3.23 |
|  |  |  |  | B+ AL705 | 3.52 |
|  | Penicillin-binding protein 2A | *lmo1892/pbpA* | Q8Y610 | Biofilm | 3.12 |
|  | UTP-glucose-1-phosphate uridylyltransferase | *lmo1078* | Q8Y840 | Biofilm | 2.07 |
|  | Glutamine-fructose-6-phosphate aminotransferase | *lmo0727/glmS* | Q8Y915 | B+ AL705 | 2.98 |
|  | Bifunctional protein GlmU | *lmo0198/glmU* | Q8YAD4 | B+ AL705 | 2.19 |
| **Coenzyme metabolism** | Uroporphyrinogen decarboxylase | *lmo2212/hemE* | Q8Y564 | Biofilm | 3.47 |
|  |  |  |  | B+ AL705 | 2.63 |
|  | 1,4-dihydroxy-2-naphthoyl-CoA synthase | *lmo1673/menB* | Q8Y6L1 | Biofilm | 2.23 |
|  | Formate-tetrahydrofolate ligase | *lmo1877/fhs* | Q8Y624 | B+ AL705 | 2.36 |
|  | NAD kinase 2 | *lmo1586/nadK2* | P65770 | B+ AL705 | 2.23 |
| **Defense mechanisms** | ABC transporter ATP-binding protein | *lmo2372* | Q8Y4R2 | Biofilm | 3.79 |
|  |  |  |  | B+ AL705 | 2.85 |
|  | ABC transporter ATP-binding protein | *lmo1636* | Q8Y6P8 | Biofilm | 2.84 |
|  | ABC transporter permease | *lmo2371* | Q8Y4R3 | B+ AL705 | 4.25 |
| **Energy production and conversion** | Pyruvate dehydrogenase subunit E1 alpha | *lmo1052/pdhA* | Q8Y865 | Biofilm | 4.97 |
|  |  |  |  | B+ AL705 | 4.79 |
|  | Pyruvate dehydrogenase subunit E1 beta | *lmo1053/pdhB* | Q8Y864 | Biofilm | 3.55 |
|  |  |  |  | B+ AL705 | 3.38 |
|  | Dihydrolipoamide acetyltransferase | *lmo1054/pdhC* | Q8Y863 | Biofilm | 3.28 |
|  |  |  |  | B+ AL705 | 2.86 |
|  | Dihydrolipoyl dehydrogenase | *lmo1055/pdhD* | Q8Y862 | Biofilm | 3.57 |
|  |  |  |  | B+ AL705 | 3.**87** |
|  | Glycerol-3-phosphate dehydrogenase | *lmo1293/glpD* | Q8Y7I4 | Biofilm | 4.93 |
|  |  |  |  | B+ AL705 | 5.71 |
|  | Glycerol kinase | *lmo1538/glpK* | Q8Y6Z2 | Biofilm | 3.53 |
|  |  |  |  | B+ AL705 | 3.50 |
|  | L-lactate dehydrogenase 1 | *lmo0210/ldh1* | P33380 | Biofilm | 3.28 |
|  |  |  |  | B+ AL705 | 2.92 |
|  | Ferredoxin-NADP reductase 2 | *lmo2390* | Q8Y4P5 | Biofilm | 3.46 |
|  |  |  |  | B+ AL705 | 2.43 |
|  | Phosphate acetyltransferase | *lmo2103/eutD* | Q8Y5G0 | Biofilm | 2.94 |
|  | NADH dehydrogenase | *lmo2389* | Q8Y4P6 | Biofilm | 2.64 |
|  |  |  |  | B+ AL705 | 2.66 |
|  | Thioredoxin reductase | *lmo2478/trxB* | O32823 | Biofilm | 2.24 |
|  |  |  |  | B+ AL705 | 2.07 |
|  | Alcohol dehydrogenase | *lmo0773* | Q8Y8W9 | Biofilm | 2.06 |
|  |  |  |  | B+ AL705 | 2.07 |
|  | Catalase | *lmo2785/kat* | Q8Y3P9 | Biofilm | 2.09 |
|  | Pyruvate-flavodoxin oxidoreductase | *lmo0829/ nifJ* | Q8Y8R6 | B+ AL705 | 2.30 |
| **Intracellular trafficking, secretion, and vesicular transport** | Protein translocase subunit SecA 1 | *lmo2510/secA1* | P47847 | Biofilm | 2.25 |
|  |  |  |  | B+ AL705 | 2.39 |
| **Lipid metabolism** | Lipase | *lmo2089* | Q8Y5H3 | Biofilm | 5.58 |
|  |  |  |  | B+ AL705 | 3.63 |
|  | Lipid kinase | *lmo1753* | Q8Y6D4 | Biofilm | 3.59 |
|  | Phosphate acyltransferase | *lmo1809/plsX* | Q8Y688 | Biofilm | 2.53 |
|  | Isoprenyl transferase | *lmo1315/uppS* | Q8Y7G6 | Biofilm | 2.48 |
|  | Enoyl-ACP (acyl-carrier-protein)-reductase | *lmo0970* | Q8Y8D5 | B+ AL705 | 3.08 |
|  | Lipoate protein ligase | *lmo0931* | Q8Y8H3 | B+ AL705 | 2.65 |
|  | NAD(P)H- dependent glycerol-3-phosphate dehydrogenase | *lmo1936/gpsA* | Q8Y5W9 | B+ AL705 | 2.01 |
| **Motility** | Flagellar motor switch protein FliM | *lmo0699/fliM* | Q8Y942 | Biofilm | 2.14 |
| **Nucleotide metabolism** | Nucleotide-binding protein | *lmo2474* | Q8Y4G9 | Biofilm | 3.51 |
|  |  |  |  | B+ AL705 | 5.64 |
|  | Acetate kinase 1 | *lmo1581/ackA1* | Q8Y6V0 | Biofilm | 2.68 |
|  |  |  |  | B+ AL705 | 3.43 |
|  | GTPase Era | *lmo1462/era* | Q8Y750 | Biofilm | 2.44 |
|  |  |  |  | B+ AL705 | 2.35 |
|  | GTPase Der | *lmo1937/engA* | Q8Y5W8 | Biofilm | 3.04 |
|  | Small ribosomal subunit biogenesis GTPase | *lmo1819/rsgA2* | Q8Y680 | Biofilm | 2.24 |
|  | GTP-binding protein | *lmo1491* | Q8Y732 | Biofilm | 2.22 |
|  | Bifunctional protein FolD | *lmo1360/folD* | Q8Y7C5 | B+ AL705 | 3.34 |
|  | Pseudouridine-5'-phosphate glycosidase | *lmo2340/psuG* | Q8Y4U2 | B+ AL705 | 3.19 |
|  | PurR family transcriptional regulator | *lmo0192* | Q8YAD9 | B+ AL705 | 2.64 |
|  | Pyrimidine-nucleoside phosphorylase | *lmo1993/pdp* | Q8Y5R3 | B+ AL705 | 2.15 |
| **Oxidoreductase activity** | Oxidoreductase G | *lmo0823* | Q8Y8S1 | Biofilm | 3.78 |
|  |  |  |  | B+ AL705 | 4.16 |
|  | CDP-abequose synthase | *lmo1694* | Q8Y6J0 | B+ AL705 | 3.48 |
|  | Aldo/keto reductase protein G | *lmo2592* | Q8Y463 | B+ AL705 | 2.13 |
| **Post-translational modification, protein turnover, and chaperones** | ABC transporter ATP-binding protein | *lmo2415* | Q8Y4M2 | Biofilm | 2.18 |
|  |  |  |  | B+ AL705 | 2.26 |
|  | Endopeptidase Clp ATP-binding chain C | *lmo0232/clpC* | Q8YAB6 | B+ AL705 | 2.28 |
| **Replication, recombination and repair** | ABC transporter ATP-binding protein | *lmo1875* | Q8Y625 | Biofilm | 5.60 |
|  |  |  |  | B+ AL705 | 3.11 |
|  | UvrABC system protein B | *lmo2489/uvrB* | Q8Y4F5 | Biofilm | 3.45 |
|  |  |  |  | B+ AL705 | 3.01 |
|  | Holliday junction ATP-dependent DNA helicase | *lmo1533/ruvA* | Q8Y6Z7 | Biofilm | 3.18 |
|  | DNA topoisomerase 4 subunit B | *lmo1286/parE* | Q8Y7J1 | Biofilm | 2.86 |
|  | ATP-dependent RNA helicase | *lmo1722* | Q8Y6G5 | Biofilm | 2.35 |
|  | UvrABC system protein A | *lmo2488/uvrA* | Q8Y4F6 | Biofilm | 2.03 |
|  | ABC transporter ATP-binding protein | lmo1431 | Q8Y770 | Biofilm | 2.00 |
|  | DNA mismatch repair protein MutL | *lmo1404/mutL* | Q8Y788 | B+ AL705 | 4.58 |
| **Signal transduction mechanisms** | Two-component response regulator | *lmo0287* | Q8YA72 | Biofilm | 2.08 |
|  | Serine phosphatase | *lmo0892/rsbU* | Q8Y8K7 | B+ AL705 | 2.92 |
|  | Two-component sensor histidine kinase | *lmo1947/resE* | Q8Y5V8 | B+ AL705 | 2.05 |
| **Transcription** | Arginine repressor | *lmo1367/argR* | Q8Y7B9 | Biofilm | 4.27 |
|  | Nucleoid occlusion protein | *lmo2794/noc* | Q8Y3P1 | Biofilm | 3.40 |
|  | Transcriptional regulator MraZ | *lmo2042/mraZ* | Q8Y5L6 | Biofilm | 3.57 |
|  |  |  |  | B+ AL705 | 4.78 |
|  | LexA repressor | *lmo1302/lexA* | Q8Y7H7 | Biofilm | 3.37 |
|  |  |  |  | B+ AL705 | 3.46 |
|  | Putative sporulation transcription regulator WhiA | *lmo2472/whiA* | Q8Y4H0 | Biofilm | 2.68 |
|  | Ribonuclease R | *lmo2449/rnr* | Q8Y4J0 | Biofilm | 2.36 |
|  | Partition protein ParB homolg | *lmo2790/parB* | Q8Y3P4 | B+ AL705 | 2.38 |
| **Translation, ribosomal structure and biogenesis** | Putative tRNA (cytidine(34)-2'-O)-methyltransferase | *lmo0935* | Q8Y8G9 | Biofilm | 6.19 |
|  | Ribosomal RNA small subunit methyltransferase H | *lmo2041/mraW* | Q8Y5L7 | Biofilm | 4.05 |
|  | Pseudouridine synthase | *lmo1843* | Q8Y657 | Biofilm | 2.47 |
|  | tRNA N6-adenosine threonylcarbamoyltransferase | *lmo2075/tsaD* | Q8Y5I7 | Biofilm | 2.41 |
|  | Elongation factor P | *lmo1355/efp* | P64032 | Biofilm | 3.53 |
|  |  |  |  | B+ AL705 | 5.87 |
|  | Ribosome maturation factor RimM | *lmo1793/rimM* | Q8Y6A2 | Biofilm | 2.56 |
|  | Ribosome-binding ATPase YchF | *lmo2779/ychF* | Q926X1 | Biofilm | 2.34 |
|  | Alanine-tRNA ligase | *lmo1504/alaS* | Q8Y722 | Biofilm | 2.15 |
|  | 50S ribosomal protein L4 | *lmo2631/rplD* | P61055 | Biofilm | 2.09 |
|  | Tyrosine-tRNA ligase | *lmo1598/tyrS* | Q8Y6T4 | Biofilm | 2.08 |
|  | Ribosomal RNA small subunit methyltransferase A | *lmo0188/ksgA* | Q8YAE2 | B+ AL705 | 3.19 |
|  | RNA-binding Sun protein | *lmo1822* | Q8Y677 | B+ AL705 | 2.35 |
|  | Valine-tRNA ligase | *lmo1552/valS* | Q8Y6X9 | B+ AL705 | 2.13 |
| **Transport of Carbohydrate and Inorganic ion** | PTS mannose transporter subunit IID | *lmo0098* | Q8YAM0 | Biofilm | 2.20 |
|  | PTS cellbiose transporter subunit IIC | *lmo2763* | Q926Y4 | B+ AL705 | 11.12 |
|  | PTS trehalose transporter subunit IIBC | *lmo1255* | Q8Y7L9 | B+ AL705 | 4.58 |
|  | PTS cellbiose transporter subunit IIA | *lmo2765* | Q8Y3R8 | B+ AL705 | 3.80 |
|  | PTS mannose transporter subunit IIC | *lmo0782* | Q8Y8W0 | B+ AL705 | 3.62 |
|  | PTS cellbiose transporter subunit IIB | *lmo2762* | Q8Y3S0 | B+ AL705 | 3.39 |
|  | PTS mannose transporter subunit IIB | *lmo0783* | Q8Y8V9 | B+ AL705 | 3.14 |
|  | Copper homeostasis protein CutC | *lmo1018* | Q8Y896 | Biofilm | 2.87 |
|  | Peptide ABC transporter permease | *lmo2194* | Q8Y580 | Biofilm | 2.60 |
|  | Fur protein | *lmo1956/fur* | Q8Y5U9 | Biofilm | 2.58 |
|  |  |  |  | B+ AL705 | 2.87 |
|  | Manganese transport system ATP-binding protein MntB | *lmo1849/mntB* | Q8Y651 | Biofilm | 2.42 |
|  |  |  |  | B+ AL705 | 3.24 |
| **Uncharacterized** | UPF0297 protein | *lmo1503* | P60357 | Biofilm | 3.59 |
|  | Hypothetical protein | *lmo2491* | Q8Y4F3 | Biofilm | 3.55 |
|  | Hypothetical protein | *lmo1401* | Q8Y791 | Biofilm | 3.21 |
|  | Hypothetical protein | *lmo0163* | Q8YAG3 | Biofilm | 2.62 |
|  | Hypothetical protein | *lmo2411* | Q928M6 | Biofilm | 2.24 |
|  | Hypothetical protein | *lmo0515* | Q8Y9L2 | Biofilm | 2.53 |
|  |  |  |  | B+ AL705 | 3.34 |
|  | Hypothetical protein | *lmo2853* | Q8Y3I3 | Biofilm | 2.09 |
|  |  |  |  | B+ AL705 | 2.44 |
|  | Hypothetical protein | *lmo0161* | Q8YAG5 | Biofilm | 2.12 |
|  |  |  |  | B+ AL705 | 2.14 |
|  | Hypothetical protein | *lmo0454* | Q8Y9R9 | B+ AL705 | 3.28 |
|  | Hypothetical protein | *lmo1966* | Q8Y5T9 | B+ AL705 | 2.74 |
|  | Hypothetical protein | *lmo0047* | Q8YAR7 | B+ AL705 | 2.43 |
|  | Hypothetical protein | *lmo0930* | Q8Y8H4 | B+ AL705 | 2.42 |
|  | Hypothetical protein | *lmo1602* | Q8Y6T1 | B+ AL705 | 2.23 |
|  | Hypothetical protein | *lmo2391* | Q8Y4P4 | B+ AL705 | 2.21 |
|  | Hypothetical protein | *lmo1257* | Q92CE7 | B+ AL705 | 2.12 |
|  | Hypothetical protein | *lmo2792* | Q8Y3P3 | B+ AL705 | 2.11 |

**Table S2.** Differentially expressed proteins by *L. monocytogenes* FBUNT in biofilm treated with lactocin AL705 compared with untreated biofilm.

| **Categories** | **Protein name** | **Locus/gene** | **N° Accession** | **Fold change** |
| --- | --- | --- | --- | --- |
| **Amino acid metabolism** | Ethanolamine ammonia-lyase large subunit | *lmo1175/eutB* | Q8Y7U5 | 13.09 |
|  | Probable glutamate decarboxylase gamma | *lmo2434* | Q8Y4K4 | 3.12 |
|  | Homoserine dehydrogenase | *lmo2547/hom* | Q8Y4A4 | 2.42 |
|  | Glutamate 5-kinase | *lmo1260/proB* | Q93Q56 | 2.25 |
| **Carbohydrate metabolism** | Alpha,alpha-phosphotrehalase | *lmo1254* | Q8Y7M0 | 14.28 |
|  | Phospho-beta-glucosidase | *lmo0319* | Q7AP84 | 9.62 |
|  | Beta-glucoside kinase | *lmo2764/bglK* | Q8Y3R9 | 6.84 |
|  | 6-phospho-beta-glucosidase | *lmo0536* | Q92EC0 | 4.62 |
|  | Probable transaldolase 1 | *lmo2743/tal1* | Q8Y3T8 | 3.89 |
|  | Beta-glucosidase | *lmo2761* | Q8Y3S1 | 3.21 |
| **Cell cycle control, cell division, chromosome partitioning** | Multidrug transporter | *lmo2463* | Q8Y4H8 | 3.53 |
| **Defense mechanisms** | ABC transporter ATP-binding protein | *lmo0607* | Q8Y9C5 | 2.31 |
| **Energy production and conversion** | Pyruvate formate-lyase-activating enzyme | *lmo1918/pflC* | P0A442 | 2.61 |
|  | Pyruvate formate-lyase | *lmo1406/pflB* | Q8Y786 | 2.55 |
|  | PflA protein | *lmo1917/pflA* | Q8Y5Y6 | 2.49 |
|  | Quinol oxidase subunit 2 | *lmo0013/qoxA* | Q8YAV0 | 2.66 |
|  | Phosphate acetyltransferase | *lmo2103*/*eutD* | Q8Y5G0 | -2.28 |
| **Lipid metabolism** | Lipase | *lmo0110* | Q8YAK8 | 2.85 |
| **Motility** | Flagellar hook protein FlgE | *lmo0697/ flgE* | Q92DV7 | -3.72 |
|  | Flagellar hook-associated protein 1 | *lmo0705/flgK* | Q8Y936 | -3.55 |
| **Nucleotide metabolism** | dITP/XTP pyrophosphatase | *lmo1239* | Q8Y7N5 | 4.48 |
|  | Pyrimidine-nucleoside phosphorylase | *lmo1993/pdp* | Q8Y5R3 | 2.68 |
| **Oxidoreductase activity** | Aldo/keto reductase | *lmo2592* | Q8Y463 | 2.36 |
| **Post-translational modification, protein turnover, and chaperones** | ATP-dependent Clp protease proteolytic subunit | *lmo1138/clpP* | Q8Y7Y1 | 2.30 |
|  | 2-cys peroxiredoxin | *lmo1604* | Q8Y6S9 | -2.48 |
| **Replication, recombination and repair** | 3\'-exo-deoxyribonuclease | *lmo1782* | Q8Y6A9 | 2.47 |
|  | Transcription-repair-coupling factor | *lmo0214/mfd* | Q8YAD0 | 2.29 |
|  | Replicative DNA helicase | *lmo0054/dnaC* | Q92FQ6 | 2.30 |
|  | ATP-dependent RNA helicase CshA | *lmo0866/cshA* | Q8Y8N0 | -2.12 |
| **Signal transduction mechanisms** | Two-component response phosphate regulator | *lmo2501/phoP* | Q8Y4E5 | 2.33 |
| **Transcription** | Transcription antiterminator | *lmo2436* | Q8Y4K2 | 3.21 |
|  | Transcripitonal regulator | *lmo0602* | Q8Y9D0 | 2.89 |
|  | Transcriptional regulator | *lmo0785* | Q8Y8V7 | 2.48 |
|  | Transcriptional regulator | *lmo0459* | Q8Y9R4 | 2.23 |
|  | Cold shock-like protein CspLA | *lmo1364/cspLA* | P0A355 | -2.22 |
| **Translation, ribosomal structure and biogenesis** | tRNA-(uracil-5-)-methyltransferase | *lmo1276/gid* | Q8Y7K1 | 2.29 |
|  | 50S ribosomal protein L6 | *lmo2617/rplF* | Q8Y444 | -2.41 |
|  | Putative tRNA (cytidine(34)-2'-O)-methyltransferase | *lmo0935* | Q8Y8G9 | -2.70 |
| **Transport of Carbohydrate and Inorganic ion** | PTS beta-glucoside transporter subunit IIABC | *lmo0027* | Q8YAT6 | 123.90 |
|  | PTS cellbiose transporter subunit IIC | *lmo2763* | Q926Y4 | 7.50 |
|  | PTS trehalose transporter subunit IIBC | *lmo1255* | Q8Y7L9 | 6.49 |
|  | PTS cellbiose transporter subunit IIA | *lmo2765* | Q8Y3R8 | 5.26 |
|  | PTS cellbiose transporter subunit IIB | *lmo2762* | Q8Y3S0 | 3.31 |
|  | PTS mannose transporter subunit IID | *lmo0098* | Q8YAM0 | -14.78 |
|  | Cation-transporting ATPase | *lmo0818* | Q8Y8S6 | 4.46 |
|  | Heavy metal-transporting ATPase | *lmo0641* | Q8Y992 | 4.45 |
|  | Sugar ABC transporter ATP-binding protein | *lmo0278* | Q8YA81 | 3.69 |
| **Vitamin metabolism** | Corrinoid adenosyltransferase | *lmo1209* | Q8Y7R2 | 4.68 |
| **Uncharacterized** | Hypothetical protein | *lmo1337* | Q8Y7E6 | 3.85 |
|  | Hypothetical protein | *lmo0794* | Q8Y8U8 | 2.54 |
|  | Hypothetical protein | *lmo1261* | Q8Y7L6 | 2.37 |
|  | Hypothetical protein | *lmo1750* | Q8Y6D7 | 2.32 |
|  | Hypothetical protein | *lmo0392* | Q92EP8 | 2.25 |
|  | Hypothetical protein | *lmo0134* | Q8YAJ1 | -4.89 |
|  | Hypothetical protein | *lmo0845* | Q8Y8Q1 | -4.51 |
|  | Hypothetical protein | *lmo0406* | Q8Y9W3 | -2.86 |
